# Three-dimensional optofluidic control using reconfigurable thermal barriers

**DOI:** 10.1038/s41566-025-01731-z

**Published:** 2025-08-08

**Authors:** Falko Schmidt, Carlos David González-Gómez, Marc Sulliger, Emilio Ruiz-Reina, Raúl A. Rica-Alarcón, Jaime Ortega Arroyo, Romain Quidant

**Affiliations:** 1 Department of Mechanical and Process Engineering, ETH Zurich, Zurich, Switzerland; 2 Universidad de Granada, Department of Applied Physics, Granada, Spain; 3 Department of Applied Physics II, University of Málaga, Málaga, Spain; 4 Multiphysics Modeling School, University of Málaga, Málaga, Spain; 5 Research Unit ‘Modeling Nature’ (MNat), Universidad de Granada, Granada, Spain

**Keywords:** Applied optics, Optical manipulation and tweezers

## Abstract

Microfluidics allows for the precise control of small sample volumes through spatial confinement and exact routing of fluids. Usually, this is achieved by physical barriers. However, the rigidity of these barriers limits flexibility in certain applications. We introduce an optofluidic approach that leverages structured light and photothermal conversion to create dynamic, reconfigurable fluidic boundaries that can be easily integrated in existing setups. This system enables the controlled manipulation of fluids and particles by generating adjustable three-dimensional thermal landscapes. We demonstrate that our reconfigurable approach replicates the functions of traditional barriers and allows real-time adjustments for tasks such as individual particle steering and size-based sorting in heterogeneous mixtures. These results highlight the potential for adaptive and multifunctional microfluidic systems in applications such as chemical synthesis, lab-on-chip devices and microbiology.

## Abstract (web summary)

A fluidic system with spatially reconfigurable hot spots generated by optical pumping of plasmonic nanorods is demonstrated, creating virtual barriers by generating local heating via photothermal conversion, for potential applications in chemical synthesis, lab-on-chip devices and microbiology.

## Main

Microfluidics is widely used in chemical and biological studies, including chemical synthesis, drug development and single-cell studies. Its high level of control over microenvironments, where a laminar-flow regime is dominant, is further enhanced by the addition of physical barriers. When strategically placed and shaped, these barriers enable comprehensive control over fluid manipulation, including pumping, mixing, splitting, merging and confinement. Additionally, they facilitate the isolation, trapping and sorting of suspended analytes like colloidal particles, cells and polymers^1^.

Common fabrication techniques for such barriers include injection moulding, lithography, additive manufacturing, micromilling and laser ablation^2–4^. Although these methods offer high resolution in both fabrication and element positioning, they are typically optimized for specific target analytes and a fixed set of manipulation tasks. This specificity reduces their ability to handle a broad spectrum of samples simultaneously, whereas the lack of tunability to adapt to varying analyte requirements further constrains their flexibility. Moreover, fixed barriers cannot be dynamically reconfigured to suit different tasks nor allow real-time experimental decision-making. Yet, tunability and reconfigurability are useful, for instance, when developing lab-on-chip devices in which multiple functionalities must be integrated to deliver a complete workflow environment.

Several strategies have been proposed to address these limitations, such as controlling flow paths in fixed pneumatic valve networks^5^, using electro-osmotic flow fields^6^, electrothermal fields^7^, magneto-hydrodynamics^8^ and hydrogels^9,10^, or creating virtual channels via hydrodynamics^11^ and thermorheological conversion^12^, among others^13^. In particular, virtual boundaries provide a flexible alternative to physical barriers by directly manipulating the fluid using body or surface forces. However, many of these methods rely on external, bulky fluidic instrumentation, require compositional changes to the liquid, or suffer from low spatial resolution and slow response times. Currently, a broadly applicable solution is still missing that provides reconfigurability with single-micrometre resolution at timescales well below 100 ms, operates without external fluid pumps and works in standard aqueous solutions.

Optofluidics with its remote and tunable control over microfluidic environments is a promising solution^14^. Among these, optothermal approaches, which exploit light-to-heat conversion, have already demonstrated precise control over synthetic particles^15–18^, cells^19,20^ and macromolecules^21,22^. Specifically, localized temperature gradients induce changes in the liquid environment such as density, viscosity or van der Waals interactions, resulting in thermo-osmosis^18^, thermophoresis^15,16,23^, natural convection^24^ and thermoviscosity^20^, all of which can induce and steer fluid flows around the heat source. Beyond heating, there are additional light-induced processes that can create microfluidic flow control^12,25^ or the development of novel display technologies^26^.

Here we introduce an optofluidic toolbox that leverages these effects by building reconfigurable virtual boundaries for particle manipulation, dynamically shaped using light. We create virtual barriers by generating local heating via photothermal conversion. Specifically, by using structured light to illuminate both surfaces of a microfluidic chamber, we generate controlled heat patterns that activate and drive fluid dynamics and particle motion throughout the volume. Through rigorous engineering of this thermal landscape, we replicate the functions of physical barriers, enabling fluidic operations such as steering, splitting and merging, as well as particle manipulations such as trapping. Combining experiments and simulations, we study the interaction of colloidal particles with the surrounding fluid patterns using different types of optofluidic barriers. Using light-based activation and rapid temperature changes, we demonstrate reconfigurability by dynamically switching the orientation of a steering barrier to control the direction of collective and individually addressed particles. Finally, we explore the use of reconfigurable virtual barriers for particle sorting by emulating an adaptive deterministic lateral displacement (DLD) assay that adjusts between particle collection and separation of varying sizes.

## Results

### Generation of localized optofluidic barriers

**Fig. 1:**
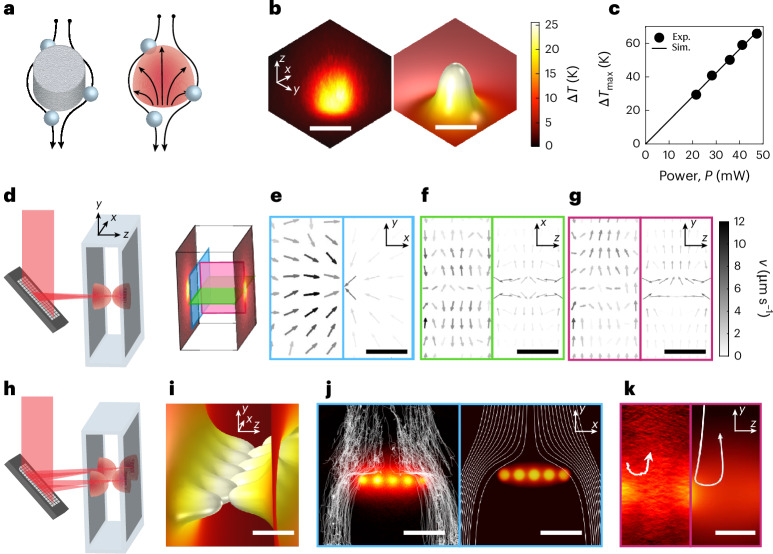
Generation of a localized optofluidic barrier. **a**, Conceptual representation of particles moving around a physical barrier made of a single pillar (left) and an optofluidic pillar (right) established by light-induced heating that creates local fluid flows (black arrows). **b**, Three-dimensional temperature profile used to create an optofluidic pillar with a single laser beam (*P* = 20 mW) on a plasmonic surface. Experimentally measured (left) and computed from simulations (right). **c**, Maximum temperature increase Δ*T*_max_ over the projected laser power *P* on the sample, experimentally measured (Exp., dots) and simulated (Sim., line) above room temperature (*T*_amb_ = 23 °C). Absolute temperatures correspond to *T* = *T*_amb_ + Δ*T*. **d**, Using an SLM, the incoming light beam is reflected and split into two, focused on each side of a microfluidic chamber (light grey) with absorbing surfaces (dark grey), leading to two opposing pillars. **e**–**g**, Comparison of experimentally measured (left) and simulated (right) fluid flows using silica tracer particles (*d*_p_ = 1.5 μm) for different planes (blue, green and purple) between two hot spots in a chamber of thickness *h* = 20 μm. **e**, Radial symmetric flows towards the centre are observed near the surface (*z* = 2 μm) due to thermo-osmotic flows. **f**, Across the chamber, flows are moving along the surfaces, converging from both sides in the middle, from where they form closed-loop flow profiles back to the surface (*y* = 25 μm). **g**, Along the vertical axis, additional convective flows push the fluid upwards (*x* = 25 μm). **h**, An optofluidic barrier is created by projecting multiple beams with alternating foci within the microfluidic chamber, demonstrating reconfigurability. **i**, Simulated optofluidic barrier shows how five hot spots on each side concatenate into each other to form a single heat barrier. **j**,**k**, Experimental (left) and simulated (right) trajectories of particles (*d*_p_ = 5 μm, white lines) sedimenting downwards (*v*_g_ = 12.7 μm s^−1^) into the barrier (temperature profile at surface shown), where they are deflected and move around (**j**), and can even be repelled upwards (**k**). Scale bars, 3 μm (**b**), 15 μm (**e**–**g** and **i**–**k**). Source data

Physical pillars are key components in traditional barriers, distorting local fluid flows to control the directionality of particles and fluid alike (Fig. 1a, left). To create virtual barriers, similar fluid flows must be induced by an external trigger, such as light. Specifically, we generate a virtual barrier by perturbing the local fluid flows with a temperature gradient near an absorbing surface (Fig. 1a, right).

We demonstrate this principle in a microfluidic chamber (thickness, *h* = 20 μm) with surfaces coated in gold nanorods (AuNRs; absorption peak, *λ*_abs_ = 780 nm), and remaining transparent for optical interrogation. At these scales, the system operates in the laminar-flow regime, ensuring that fluid mixing occurs predominantly by diffusion. Illumination with a single light beam, resonant with the absorbing surface, locally increases the temperature Δ*T* of the surrounding medium relative to the ambient temperature *T*_amb_ (Fig. 1b). By increasing the applied laser power *P*, Δ*T* increases linearly (Fig. 1c).

The beam (*λ*_light_ = 807 nm) is then shaped using a spatial light modulator (SLM) to focus simultaneously on both sides of the chamber coated with AuNRs (Fig. 1d). Therefore, we induce thermally driven phenomena, such as thermo-osmosis, thermophoresis and natural convection (Supplementary Fig. 1). These thermal effects drive fluid flows and particle motion within the entire volume of the chamber (Fig. 1e–g).

To characterize the system, we measure the resulting temperature profiles and complex flow patterns using optical diffraction tomography (ODT) and digital holographic microscopy, respectively, and compare the results with simulations. Near the surfaces, temperature increases by Δ*T*_max_ = 20 K are reached in less than 1 ms (ref. ^27^) inducing thermo-osmotic flows^18^ directed radially inwards (Fig. 1e). These inward flows converge towards the centre of the chamber, where they meet and then redirect outwards, forming closed loops (Fig. 1f). Thermo-osmotic flows occur due to changes in the van der Waals interaction at the surface–liquid interface under a temperature gradient ∇*T*; therefore, they are mainly localized near the surfaces (*z* < 10 μm). In addition to ∇*T*, their strength depends on materials properties such as thermal conductivity *κ* (Supplementary Fig. 1). Thermophoresis, also dependent on ∇*T*, occurs due to changes in the interaction of the particle with the surrounding liquid. Natural convection is induced by density changes due to ∇*T*, leading to the buoyancy of suspended particles. It is observed along the gravitational axis *y* (Fig. 1g) and strongly depends on chamber thickness *h*, becoming predominant for *h* ≥ 50 μm (Supplementary Fig. 2). Together, these thermally induced flow fields disturb the otherwise laminar-flow fields of sedimenting or pressure-driven particles, creating an effective obstacle for the fluid that we refer to as optofluidic pillar.

Since heat sources are additive, they can be combined to create a fluidic profile mimicking a physical barrier composed of individual pillars. By spatially and temporally modulating the AuNR-resonant light, a reconfigurable optofluidic barrier made of individual pillars across the microfluidic chamber (Fig. 1h) can be created. To generate an efficient optofluidic barrier, we alternated heat sources between both surfaces (Fig. 1i). Such double-sided heating is crucial, as sedimentation or pressure-driven particles would otherwise escape along the larger gaps in the temperature profile (Supplementary Fig. 3a,b and Supplementary Video 1).

Analogous to a physical barrier, the virtual barrier deflects any approaching particle, due to the presence of strong fluid flows (Fig. 1j,k and Supplementary Video 2).

Although thermo-osmosis primarily governs the particle trajectories (Supplementary Fig. 1c,d), thermophoretic contributions are also notable (Supplementary Fig. 1g,h). Previous studies^28,29^ confirm that our dielectric particles (SiO_2_, microParticles) are weakly thermophobic. Consequently, these particles move away from high-temperature regions with a thermal drift velocity *D*_T_ = 8.7 μm s^−1^ K^−1^, enhancing the barrier’s performance. Our simulations agree well with experimental observations (Supplementary Fig. 1k) and confirm the presence of all three contributions with only slight discrepancies attributed to the difficulty in accurately determining the thermophoretic coefficient^23^ and to neglect Brownian motion contributions in the simulations (Methods). Other non-thermal effects, such as optical forces, are negligible, as the laser beams used for heating were focused just outside the chamber, with only sedimentation (*v*_g_ = 1.1 μm s^−1^; Methods) remaining (Supplementary Fig. 1j and ref. ^28^). We characterized the barrier’s efficiency by quantifying the fraction of particles (*d*_p_ = 5 μm) passing through the barrier given a certain particle flux (Methods). The efficiency predominantly depends on the spatial configuration of our heat sources, which, for our barrier, is given by the distance between each pillar (Supplementary Fig. 3c) and the induced temperature differences Δ*T*_max_. The best performance was found for pillar distances ≤13.3 μm and Δ*T*_max_ ≥ 16.2 K. For a sedimentation-driven system, the particle size *d*_p_ determines the sedimentation speed^30^ and, thus, the required Δ*T* for compensation.

So far, we have utilized sedimentation to passively drive the particles towards the barrier. Alternatively, pressure pumps can be used with the advantage of tuning the particles’ flow speed independent of their size. Under a pressure-driven flow, we show flow speeds up to 12 μm s^−1^ and retention of the full functionality of the barrier, defined as a 90% deflection efficiency (Supplementary Fig. 4). Higher velocities up to 80 μm s^−1^ are possible with chamber heights of *h* = 37 μm due to increased natural convection utilizing temperature differences up to 120 K (Supplementary Fig. 2).

Optofluidic barriers can be actively tuned through laser power adjustment and heat source shaping, eliminating the need for precise prior knowledge of the present thermal effects. By monitoring specimen interactions with the barrier, parameters can be tuned in real time, ensuring optimal performance.

**Fig. 2:**
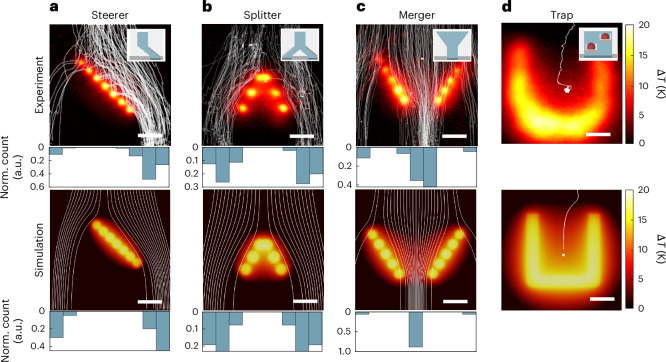
Types of optofluidic barriers. Experimental images (top) of heat-induced barriers with particle trajectories (*d*_p_ = 5 μm, white lines) sedimenting downwards at *v*_g_ = 12.7 μm s^−1^, accompanied by three-dimensional sketches of the equivalent microfluidic actuators (insets). Simulated temperature profiles (bottom) of the barriers with particle trajectories (white). **a**, A steerer, created by a single barrier tilted at –45°, deflects particles to the right, similar to the effect of a tilted microfluidic channel. **b**, A splitter, created by two tilted barriers, evenly divides the particle flow into two, resembling a Y-shaped microfluidic channel with two outlets. **c**, A merger directs particles to converge at the centre, akin to a narrowing channel. **d**, A U-shaped barrier traps a particle entering from the top, confining it from both sides to a single equilibrium point, similar to micropockets in traditional fluidic channels. Norm., normalized. Scale bars, 10 μm. Source data

**Fig. 3:**
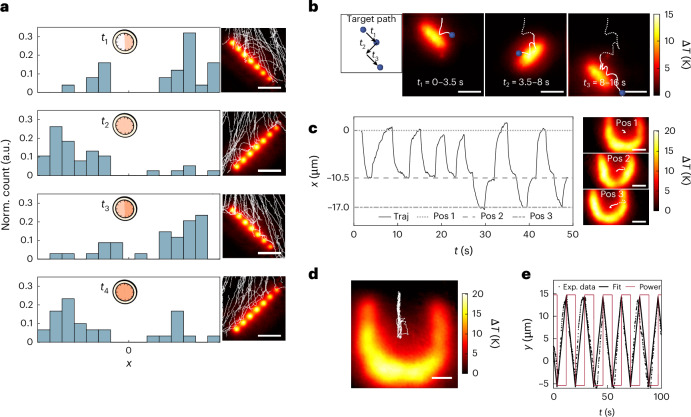
Reconfigurability of optofluidic barriers. **a**, A single steerer barrier is periodically switched between two orientations (–45° for *t*_1_ and *t*_3_, and 45° for *t*_2_ and *t*_4_) every 30 s (clock), demonstrating that particles (*d*_p_ = 5 μm, white lines) sedimenting downwards at *v*_g_ = 12.7 μm s^−1^ can be deflected left and right over time, as shown by the shifts in distribution in the histograms (blue). **b**, By defining a target path for a sedimenting particle, the steerer barrier is dynamically positioned and reoriented to guide the particle along the desired trajectory. **c**, Trapping position of a particle (left) is laterally shifted between three predefined positions (Pos 1–3) by translating the U-shaped barrier, visible in the particle’s *x*-axis trajectory (Traj). Example trajectories and temperature plot (right) for *t* = 25–30 s. **d**, Vertical shifts in the trapped particle’s position are achieved by adjusting the laser input power. **e**, A rectangular laser power signal (red) between *P* = 95 mW and 135 mW induces a triangular particle position response (black dots) as the particle transitions between two equilibrium points, with the position fitted by a triangular function with a 17-s period. Scale bars, 25 μm (**a**–**c**); 10 μm (**d**). Source data

To demonstrate full reconfigurability in lab-on-chip devices, the utilized method must (1) be able to set a desired function and (2) execute changes in real time^13^. We showcase both capabilities by generating barriers with diverse functionalities for particle manipulation (Fig. 2), and dynamically switching between configurations operating at either the ensemble or single-particle level via real-time feedback (Fig. 3). We achieve these functionalities by engineering the thermal landscape through projected light patterns that generate optofluidic barriers of varying length, orientation and composition. We focused our particle and fluid characterization on three basic barrier types, which mimic the core functions of classical microfluidics (Fig. 2a–d, insets): steerer (Fig. 2a and Supplementary Video 3), splitter (Fig. 2b and Supplementary Video 4) and merger (Fig. 2c and Supplementary Video 5).

For the steerer, we experimentally created a –45°-tilted barrier that deflects particles towards the right (Fig. 2a), mimicking the microfluidic design of an inclined channel (Fig. 2a, inset). Simulations show similar steering behaviour, with a higher concentration of particles emerging on the right (Fig. 2a, histogram). For particles far left of the barrier, thermophoresis drove them further away, providing an additional tunable parameter via particle surface functionalization^29^. By symmetry, a 45°-tilted barrier steers the particles to the left (Fig. 3a). In microfluidic chambers with *h* ≥ 50 μm, where thermo-osmosis plays a weaker role, a steerer can be engineered from the predominant natural convection contributions instead (Supplementary Fig. 5).

To create a splitter that divides the particle flux evenly between left and right sections, we combined two tilted barriers (Fig. 2b). This design resembles a Y-channel microfluidic architecture made of one input and two output channels (Fig. 2b, inset). The slight asymmetry observed in experiments is attributed to the limited number of recorded trajectories (Fig. 2b, histogram).

Conversely, we created a merger by inverting the splitter design, leaving a gap of 20 μm at the bottom to focus particles from both sides towards the centre (Fig. 2c), physically mimicking a narrowing channel (Fig. 2c, inset). Beyond these linear barrier designs, additional functionalities, such as trapping, emerge when more complex patterns are used. For instance, a U-shaped barrier allows particles to enter from the top, whereas confining them from all other directions (Fig. 2d and Supplementary Video 6). Such functionality is akin to micropockets in flow channels (Fig. 2d, inset) that allow, for instance, long-term studies of trapped analytes^31,32^.

This specific example demonstrates that more complex workflows can be delivered by superposing multiple barrier types (Supplementary Fig. 6). Unlike physical barriers, optofluidic counterparts offer greater control via independent interactions of temperature gradients with fluids (thermo-osmosis and natural convection) and particles (thermophoresis). These added degrees of freedom enable the precise manipulation of particle motion, concentration and positioning, enhancing applications like analyte detection and synthesis reactions.

Since temperature gradients on the microscale are induced within milliseconds^27^, these optofluidic barriers can be reconfigured in real time to meet changing experimental conditions and needs (Fig. 3). Using a liquid-crystal-on-silicon-based SLM (60-Hz refresh rate), we dynamically switch between optofluidic barriers. As an example of passive reconfigurability, we periodically flipped a steerer barrier, altering the spatial distribution of particles from right to left (Fig. 3a and Supplementary Video 7). For statistical accuracy, each steerer orientation was maintained for 30 s; however, much faster switching is possible, as shown by the targeted steering of a single particle (Fig. 3b and Supplementary Video 8). To achieve active feedback at the single-particle level, we defined a target path and projected a barrier to induce the desired response based on real-time particle tracking (Methods). For example, a steerer with a –45° tilt directs the particle downwards and to the right, whereas the opposite tilt moves the particle left and down. This process strongly depends on the particle’s individual interaction with the barrier, as its exact position cannot be precisely predicted in the presence of random Brownian motion. Another example was demonstrated by shifting the equilibrium position of a single particle within an optofluidic trap (Fig. 3c–e and Supplementary Video 9). Here shifting the position of the U-shaped barrier tuned the particle’s lateral position (Fig. 3c), and adjusting the input laser power provided vertical control (Fig. 3d,e). The latter example illustrates reconfigurability beyond spatially reshaping the thermal environment; instead, the particle’s motion is controlled by tuning its buoyancy, which is directly determined by the amount of power converted to heat (Fig. 3e). Altogether these results demonstrate that optofluidic barriers not only emulate functionalities associated with conventional barriers but also deliver added features such as feedback-based positional control owing to the tunability and reconfigurability of thermal energy landscapes.

#### Particle steering in periodic arrays

**Fig. 4:**
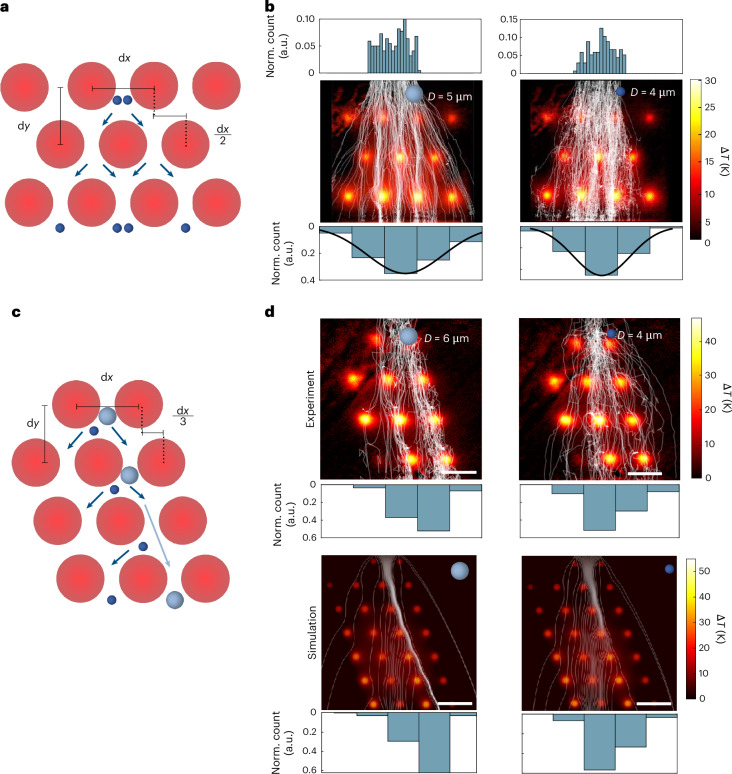
Reconfigurable particle steering in periodic patterns. **a**, Periodic arrangement of optofluidic pillars alters the initial particle distribution, showing resemblance to a Galton board experiment. **b**, Particles move from top to bottom through the periodic optofluidic pattern with an irregular starting distribution, resulting in a binomial distribution of particle positions at the bottom (after normalization), which approaches a normal distribution (black line) for many trials (>200). This behaviour is independent of particle size, demonstrated here for diameters of *d*_p_ = 5 μm sedimenting at *v*_g_ = 12.7 μm s^−1^ (d*x* = 27 μm and d*y* = 30 μm) and *d*_p_ = 4 μm at *v*_g_ = 8.2 μm s^−1^ (d*x* = 24 μm and d*y* = 27 μm). **c**, By laterally shifting consecutive rows of pillars by d*x*/3, the pattern of the particle output distribution can be skewed, bearing similarities to DLD experiments for particle sorting. **d**, Larger particles (*d*_p_ = 6 μm, *v*_g_ = 18.4 μm s^−1^) are displaced up to 52% in experiments (60% in simulations) towards the bottom-right segment, whereas smaller particles (*d*_p_ = 4 μm, *v*_g_ = 8.2 μm s^−1^) are less deflected (31% experiments, 35% simulations), effectively separating the two species. This behaviour is observed in both experiments and simulations. Scale bars, 10 μm (experiment); 20 μm (simulations). Source data

To demonstrate the potential of reconfigurable optofluidic barriers for advanced particle manipulation, we investigated a two-dimensional array of optothermal pillars and test it for particle sorting. By tuning the size and spacing of the optofluidic pillars, we enhanced the steering efficiency for particles of various sizes. Initially, we arranged the pillars with each row shifted by half the interpillar distance, d*x*/2, allowing free adjustment of the spacing along *x* and *y* (Fig. 4a). This pillar configuration, reminiscent of a microscopic Galton board, showcases how optofluidic barriers guide particles into distinct distributions.

As particles traverse the periodic array, successive interactions cause lateral displacement, leading to a symmetric output distribution. After tracking 200 individual particles, we find that the distribution approaches a normal distribution (Fig. 4b). Deviations from the ideal binomial distribution expected in mechanical Galton boards probably arise from Brownian motion and local variations in light intensity and absorption, resulting in varying Δ*T* across pillars.

Since particle–pillar interactions depend on pillar spacing, fixed patterns cannot effectively steer particles of different sizes. By tuning pillar spacing (for example, d*x* = 27 μm and d*y* = 30 μm for *d*_p_ = 5 μm, and d*x* = 24 μm and d*y* = 27 μm for *d*_p_ = 4 μm), we achieve comparable distributions across sizes.

Building on this, we further adapted the two-dimensional array to achieve directional particle steering based on size. By introducing a slight lateral offset between successive rows (d*x*/3), we generated size-dependent distributions within the same structure in which larger particles are displaced laterally with a rightward bias whereas smaller ones follow the original flow path (Fig. 4c), inspired by DLD assays^33^. This size sorting mechanism is independent of specific particle–liquid interactions, contrary to other thermal sieving techniques based on thermophoresis^16,34^. Experimentally, we show the principle of particle sorting for a sample composed of particles with *d*_p_ = 6 μm and *d*_p_ = 4 μm using an optofluidic pillar pattern with the following parameters: d*x* = 17.5 μm and d*y* = 20 μm (Fig. 4c). Larger particles exhibited a right bias (~60%), whereas smaller particles had a left bias (~69%), well in agreement with the DLD design (Fig. 4d). For particles below a threshold size (*d*_p_ ≈ 3.5 μm), the separation mechanism shifts as the balance between buoyancy and sedimentation changes (Supplementary Fig. 7 for *d*_p_ = 2.8 and 1.5 μm). Though our experiments were not optimized for high separation efficiency, we achieved a notable shift in size distributions within a working length of <100 μm, providing an alternative method to conventional DLD systems^33^. We used simulations to first validate our experiments, observing good agreement for larger particles (*d*_p_ = 6 μm) deviating to the right between the observations in experiments (*p*_exp_ = 0.52) and the predictions made in simulations (*p*_sim_ = 0.61). The small difference probably arises due to the absence of any Brownian motion in simulations. We then extrapolated beyond the experimental field of view (Fig. 4d). We observed that smaller particles deviate left just before the third row, whereas larger ones continue downstream. Adjusting the temperature profile within the DLD array improved the sorting efficiency by exploiting thermophobic behaviour, guiding large particles along the designed gap in the pattern and enhancing the lateral displacement of smaller ones (Supplementary Fig. 8). To better understand how the thermal landscape of the DLD assay affects particle deflection depending on their size, we performed a sweep for the pillar spacings d*x* and d*y*, and found a distinct regime for which only the larger size is deflected (Supplementary Fig. 9). This highlights the importance of thermophoretic effects for sorting, providing additional criteria beyond particle size.

These results demonstrate that combining thermally tunable fields with optofluidic barriers improves the sorting precision and efficiency, particularly for closely sized particle mixtures, compared with static DLD designs for compact microfluidic systems.

## Discussion

We have demonstrated that light-induced heating within a microfluidic chamber can create dynamically tunable optofluidic barriers. Our results show that these barriers not only manipulate particle flow similar to physical ones but can be further engineered to perform more complex microfluidic tasks such as steering, merging, splitting and trapping, all in a reconfigurable manner. These optofluidic barriers can be adjusted in real time to perform multiple tasks within a single microenvironment. Crucially, the adaptability and reconfigurability of our approach remain unaffected by the specific thermal effects at play. By leveraging a feedback loop to dynamically tune the thermal field, the interaction strength and particle behaviour can be precisely adjusted without requiring prior knowledge of the underlying thermal dependencies or specimen properties.

Despite the use of high-temperature gradients Δ*T*_max_ in our experiments, the method may be applicable to a broad range of analytes, including particles and potentially cells and macromolecules, although further studies are needed to assess their viability and keeping the absolute temperatures *T* = Δ*T*_max_ + *T*_amb_ within conditions of biological relevance^17,21,28^.

Although our platform is based on thermally induced fluid flows to generate virtual boundaries, the principle of using spatial light modulation to induce localized temperature changes illustratively demonstrated here is applicable to other temperature-dependent processes in which barriers can be reversibly formed with only minor temperature changes (Δ*T*_max_ < 1 K; refs. ^9,10,35^).

The ability to rapidly induce heat allows for real-time adjustments with feedback loops, which provides a promising approach for reconfigurable microfluidics.

## Methods

### Sample preparation

Our microfluidic chip comprised two coverslips separated by a silicone spacer of variable height (*h* = 20 μm and *h* = 50 μm). The coverslips (borosilicate; thickness, 150 μm; thermal conductivity *κ* = 1.2 W K^−1^ m^−1^) were coated with a homogeneous layer of synthesized AuNRs (13 × 46 nm^2^) with localized surface plasmon resonance at 780 nm (ref. ^28^). To attach AuNRs, coverslips with a thickness of 170 μm were cleaned, immersed in EtOH and 2% APTES for 1 h, and then rinsed with EtOH and dried at 100 °C for 20 min. The AuNR solution was drop cast, incubated for 20–40 min, and then rinsed and dried with N_2_ gas. The chamber was assembled by placing a silicone spacer on one coverslip, adding the particle solution, and sealing with the second one.

As the particle solution, we used SiO_2_ spheres (microParticles) in deionized water. For tracing fluid flows, *d*_p_ = 1.0 μm was used, and for optofluidic barrier experiments, *d*_p_ values of 1.5 μm, 2.8 μm, 4.4 μm, 5.2 μm and 6.2 μm were used.

### Digital holographic microscopy

We built an off-axis digital holographic microscope (Supplementary Fig. 10, blue-beam path) using a 465-nm laser (LaserTack) divided into two paths via a 50:50 fibre splitter. The imaging path, focused on the sample using an objective (Olympus LM Plan FI ×50/0.5), is collected by a high-numerical-aperture objective (Olympus, planN ×40/0.65) and projected onto a complementary metal–oxide–semiconductor camera (Basler, aca1920). A reference path matches the imaging path’s optical length, entering the camera at a slight angle, producing holograms for particle tracking and refractive-index measurement. The field of view is 100 × 100 μm^2^ (ref. ^28^).

### ODT

We utilized ODT for three-dimensional temperature mapping by measuring the complex refractive index of the sample (Supplementary Fig. 10, blue-beam path). The refractive-index profile was reconstructed from multiple phase and amplitude images taken at varying angles of illumination. The angle was changed using a rotating wedge prism, and each phase and amplitude image was recorded using the off-axis holographic microscope. To enhance the signal-to-noise ratio, we averaged 50 complex electric-field maps for each angle in a pump–probe configuration. The scattered electric field for each angle was calculated using the Rytov approximation and combined in the corresponding Ewald sphere. The inverse Fourier transform of this sphere yields the three-dimensional refractive index profile. From this, the temperature distribution is calculated using known expansion coefficients of water, as detailed in ref. ^36^ and further explained in ref. ^27^.

### Spatial light modulation

Heating of the sample was achieved using a 1-W continuous-wave laser at *λ* = 807 nm (Roithner Laser, RLT-MDL-808 DL20296). The laser beam was expanded five times using a telescope and directed to a liquid-crystal-phase-only SLM (Hamamatsu). By controlling the phase mask on the SLM through a computer, various light patterns could be generated, allowing full control over the beam’s position and focus (Supplementary Fig. 10, red-beam path). The phase mask was calculated using a custom MATLAB code (Version 2023a) based on the iterative Gerchberg–Saxton algorithm. A filter was used to block the zeroth-order beam, allowing only the tunable first-order beam to pass. This beam was projected through a 4*f* system and focused onto the sample using a high-numerical-aperture objective (Olympus, planN ×40/0.65), creating localized heating in specific regions of the microfluidic chamber.

### Particle tracking

To trace the fluid flow in the microfluidic chip, we tracked the movement of small SiO_2_ tracer particles (*d*_p_ = 1 μm) suspended in the liquid. Using the holograms recorded by our custom setup, we extracted the phase and amplitude information to locate and track particles in three dimensions over time. The process involved Fourier transforming the images, isolating the desired frequency domain and applying filters to highlight the particles as bright spots against a dark background. The particle positions in the *x* and *y* planes were determined using subpixel accuracy through radial symmetry methods^37^. By contrast, the *z* positions were obtained by projecting the Fourier-transformed image onto various planes until the intensity maximum was found, corresponding to the particle’s depth. The trajectories of individual particles were constructed by connecting their positions over time using MATLAB routines. Flow tracing was performed by defining voxels in the measured sample volume and calculating the average velocity (direction and magnitude) of particles passing through each voxel. These data were used to visualize the fluid flow as arrow plots (Fig. 1e–g), with additional details on trajectory analysis provided in ref. ^28^.

### Microfluidic setup

For our pressure-driven experiments (Supplementary Fig. 4), we utilized two types of microfluidic chip. First, we used a custom-made chip made of polydimethylsiloxane with a single channel (width, 200 μm; height, 6 μm) covered by a glass slide (borosilicate; thickness, 170 μm) to test the lower limit of the height dimension but were limited by the maximum applicable Δ*T* before polydimethylsiloxane started to visually deform. We, thus, switched to a commercial microfluidic chip made of glass with a single channel (width, 100 μm; height, 37 μm; Nr. 10001444, microfluidic ChipShop). To establish a physical interface with the glass chip, two polydimethylsiloxane blocks with a single central inlet hole were aligned and bonded to the chip inlet/outlet after exposure to oxygen plasma. Regarding the inlet, the microfluidic chip was connected to Tygon tubing (*ø*_in_ = 0.508 mm and *ø*_out_ = 1.524 mm; Saint-Gobain) via a bent metal pin (Tube AISI 304, 0.65/0.35 × 17.5 mm^2^; Unimed SA). This tubing was subsequently linked to pressure controllers (LU-FEZ-1000, Fluigent) using a dispensing tip (LuerLock 1/2 inch; inner diameter, 0.34 mm; GONANO Dosiertechnik) with a male LuerLock-to-Barb Adapter (Darwin Microfluidics) and 4 × 2.5 mm^2^ polyurethane tubing (917-2407, RS Pro). At the outlet, a single tubing was connected to a reservoir.

### COMSOL Multiphysics simulations

We have performed several numerical simulations to model the optofluidic behaviour observed in experiments, based on previous models^38^. For this purpose, we used the finite element analysis software COMSOL Multiphysics v. 6.2 to solve the equations for coupled heat transfer and fluid flow, as well as to couple these fields with the particle-tracing equations. In the following, we describe the methodology used to perform the simulations.

First, we construct the geometry by considering a box with a square base of 500 μm required to fully visualize the generated vortices. The thickness of the simulated chamber was varied according to experiments between 20 μm and 50 μm, whereas the simulated glass coverslip was chosen to be 150 μm, important for correctly calculating the heat dissipation. As the heat source, we considered a simplified model consisting of a circular hot spot of radius 4 μm, mimicking the absorption cross-section of a laser beam in the experiments. Depending on the characteristics of each barrier configuration, we introduced multiple hot spots.

We used material properties from the COMSOL Multiphysics library, including water as the liquid and silica as glass. To simulate the gold nanoparticle agglomerate on a glass coverslip, we introduced an effective material condition, assigning 60% of the weight to gold and 40% to water.

To solve the coupled equations for the temperature distribution and flow field, we introduced the Heat Transfer in Fluids and Laminar Flow physics interfaces into the model, as well as their coupling via Nonisothermal Flow Multiphysics, including the convective term in heat transfer and the buoyancy body force, and Particle Tracing for Fluid Flow physics interface. Since particles do not notably alter the flow field, the previously mentioned models were calculated separately, reducing the computational load.

We applied the Heat Transfer in Fluids physics interface in all domains (fluid and solid coverslips). A fixed temperature boundary condition of 298.15 K was considered for all external boundaries, except for the coverslips boundaries open to air, where we defined a heat flux boundary condition to account for external natural convection (here present as natural convection). We also introduced a thin-layer node with a non-stratified shell type to take into account the heat transport through the gold nanoparticle agglomerate deposited in the inner coverslip surfaces. Finally, we used boundary heat source conditions for laser heating at the hot spots, assuming a uniform heat distribution within the spots and adjusting the heat generation value according to the experimental temperature measurements.

The Laminar Flow physics interface was exclusively applied in the liquid domain, utilizing a weakly compressible flow physical model to account for natural convection. We established an open boundary for the lateral walls, compensating for hydrostatic pressure. To replicate thermo-osmotic flows in experiments, we implemented a slip velocity wall boundary condition for the heated walls by incorporating thermal creep and adjusting the thermal slip coefficient to match the velocity values observed in experiments. The equation for this boundary condition is

1 $${\bf{u}}({\bf{r}})=\frac{{\sigma }_{{\rm{T}}}\mu }{{\rho }_{{\rm{l}}}({\bf{r}})T({\bf{r}})}{\nabla }_{{\rm{t}}}T({\bf{r}}),$$

where *μ* is the dynamic viscosity of the fluid and *σ*_T_ is the thermal slip coefficient. Also, *ρ*_l_(**r**) is the density of the fluid, **u**(**r**) is the fluid velocity, *T*(**r**) is the local temperature and ∇_t_*T*(**r**) is the tangential gradient of the temperature at the boundary, where **r** specifies the local position at the boundary. Note that there is a dependence of the fluid density on position **r**, which is due to the local increase in temperature. This is also true in the Navier–Stokes and heat transfer equations below; therefore, we are taking into account the natural convection of the fluid due to temperature density fluctuations, as well as the boundary thermo-osmotic flow.

When heating only one side of the chamber, we applied a no-slip wall boundary condition to the opposite wall, which constrains the fluid velocity to zero at the wall.

Given the low Reynolds number of tracer particles, any inertial terms were neglected. We assumed the density of the silica particles to be 1,850 kg m^−3^ and their thermal conductivity to be 0.15 W mK^−1^. The particle starting positions were defined using a Release from Grid node. In most simulations, we utilized 30 particles with a spacing of 2.5 μm between them. The particles were positioned at half the height of the chamber, and the *y* position was adjusted based on the specific experiment. We accounted for drag, gravitational and thermophoretic forces acting on the particles.

Regions of high temperature required special mesh control to correctly calculate and visualize the temperature gradient and thermo-osmotic flows near the wall. We finely meshed the boundaries associated with the hot spots and ensured, through a mesh dependency analysis, that the final numerical results were independent of the mesh size, with a relative error of less than 5%. To test the sensitivity of our system, we reduced the minimum mesh size from 1.375 μm down to 0.138 μm, leading to a maximum error of around 1.7%. The discretization error is particularly lower in regions far from the heat sources. In stationary studies, we used a fully coupled approach, using the algebraic multigrid method, which uses a generalized minimal residual method solver with a relative tolerance of 10^−3^. Moreover, for the time-dependent studies, we have also used a fully coupled approach, using an iterative method, which also uses a generalized minimal residual method solver with a relative tolerance of 10^−5^.

We obtained the numerical solutions in two steps. First, we conducted a stationary coupled study for Heat Transfer in Fluids and Laminar Flow physics interfaces. Second, we performed a time-dependent analysis, which utilized the flow-field solution from the first step. Our simulations solved the strongly coupled heat transfer (equation (2)) and Navier–Stokes (equation (3)) equations, taking into account the thermo-osmotic boundary condition (equation (5)), for calculating the temperature and flow velocity fields, respectively:

2 $${\rho }_{{\rm{l}}}({\bf{r}}){c}_{{\rm{p}}}{\bf{u}}({\bf{r}})\cdot \nabla T({\bf{r}})-\kappa {\nabla }^{2}T({\bf{r}})=q({\bf{r}}),$$

3 $$\begin{array}{c}\nabla\left[p({\bf{r}}){\bf{I}}-\mu (\nabla {\bf{u}}({\bf{r}})+{(\nabla {\bf{u}}({\bf{r}}))}^{{\rm{T}}})\right]={\rho }_{{\rm{l}}}({\bf{r}}){\bf{g}}\\ \nabla \cdot (\;{\rho }_{{\rm{l}}}({\bf{r}}){\bf{u}}({\bf{r}}))=0\end{array},$$

where *q*(**r**) is the heat source due to laser absorption. *c*_p_, *κ* and *μ* are the specific heat capacity, thermal conductivity and dynamic viscosity of the aqueous solution, respectively. *T*(**r**), **u**(**r**) and *p*(**r**) are the spatial temperature, fluid velocity and fluid pressure fields, respectively. **I** is the constant 3 × 3 identity matrix and the superscript T denotes matrix transposition.

In the second step, COMSOL Multiphysics computed the particle positions using a first-order formulation that solves only for the particle positions:

4 $$\frac{{\rm{d}}{{\bf{q}}}_{{\rm{p}}}}{{\rm{d}}t}={\bf{u}}({{\bf{q}}}_{{\rm{p}}})+\frac{{\tau }_{{\rm{p}}}}{{m}_{{\rm{p}}}}{{\bf{F}}}_{{\rm{ext}}}({{\bf{q}}}_{{\rm{p}}}),$$

where **q**_p_ is the instantaneous position of the particle, and **u**(**q**_p_) is the flow velocity **u**(**r**) computed previously from equations (1)–(3) at the instantaneous particle position. In the same expression, *m*_p_ and ${\tau }_{{\rm{p}}}={\rho }_{{\rm{p}}}{d}_{{\rm{p}}}^{\;2}/18\mu$ are the mass of the particle and the particle velocity response time, respectively. *ρ*_p_ and *d*_p_ are the density and diameter of the particle, respectively. **F**_ext_ corresponds to the sum of the forces applied to the particles. Rather than solving for the particle velocity or momentum, this formulation defines the velocity based on the assumption that the drag force perfectly counterbalances all the other applied forces on the particle at any instant in time. It is appropriate when the timescale over which the particles accelerate in the fluid is very small compared with the total simulation time.

In our case,

5 $${{\bf{F}}}_{{\rm{ext}}}({{\bf{q}}}_{{\rm{p}}})={{\bf{F}}}_{{\rm{D}}}({{\bf{q}}}_{{\rm{p}}})+{{\bf{F}}}_{{\rm{g}}}({{\bf{q}}}_{{\rm{p}}})+{{\bf{F}}}_{{\rm{th}}-{\rm{ph}}}({{\bf{q}}}_{{\rm{p}}}),$$

where **F**_D_, **F**_g_ and **F**_th–ph_ are the drag, gravity and thermophoretic forces applied to the particles, respectively. They can be correspondingly expressed as

6 $${{\bf{F}}}_{{\rm{D}}}({{\bf{q}}}_{{\rm{p}}})=\frac{1}{{\tau }_{{\rm{p}}}}{m}_{{\rm{p}}}({\bf{u}}({{\bf{q}}}_{{\rm{p}}})-{\bf{v}}),$$

7 $${{\bf{F}}}_{{\rm{g}}}({{\bf{q}}}_{{\rm{p}}})={m}_{{\rm{p}}}{\bf{g}}\frac{{\rho }_{{\rm{p}}}-{\rho }_{{\rm{l}}}({{\bf{q}}}_{{\rm{p}}})}{{\rho }_{{\rm{p}}}},$$

8 $${{\bf{F}}}_{{\rm{th}}-{\rm{ph}}}({{\bf{q}}}_{{\rm{p}}})=-\frac{6\uppi {d}_{{\rm{p}}}^{\;2}{\mu }^{2}{c}_{{\rm{s}}}\varLambda \nabla T({{\bf{q}}}_{{\rm{p}}})}{{\rho }_{{\rm{l}}}({{\bf{q}}}_{{\rm{p}}})(2\varLambda +1)T({{\bf{q}}}_{{\rm{p}}})},$$

where *Λ* = *κ*/*κ*_p_, *κ*_p_ is the particle thermal conductivity and **v** is the velocity of the particle. In equation (8), *T*(**q**_p_) is the temperature field *T*(**r**) calculated previously in equations (1)–(3) at the instantaneous particle position. Also, *c*_s_ is the thermophoretic correction factor. We adjusted the value of this factor to align the numerical results with the experimental results.

The thermophoretic force acting on the particles directly depends on the temperature gradient in the fluid (calculated in the first step) at the position where the particle is located. It is not an external force acting on the whole system (fluid + particles), but it is an external force if we consider only its influence on the motion of the particles, as we do in the second step. A system exhibiting thermophoretic drift of the colloids can be considered force free (thermophoretic force is an internal force). However, here we do not consider the influence of the thermophoretic force on the fluid flow, because we implicitly disregard it by dividing the calculation into two parts: first, the velocity and temperature distributions of the fluid, driven mainly by thermo-osmosis and natural convection; second, the motion of the particles under these fields, driven by drag, natural convection and thermophoretic forces.

To compute the number of particles in the histograms, we defined nine bins in the liquid domain, each of them 10 μm wide using Particle Counter domain nodes.

For modelling the DLD experiments, we considered different configurations for hot-spot diameters; powers; and distance separations d*x* and d*y* along the *x* and *y* axes, respectively. Here we launched 30 particles, placed on top of the DLD structure, from the centre of the left hot spot to the centre of the right hot spot (varying the particle interdistance depending on the d*x* value). For these simulations, we reduced the bins to five. We obtained optimal deflection results for particles with *d*_p_ = 6 μm and separations d*x* = 33 μm and d*y* = 30 μm. In this case, we assigned different heating powers to the hot spots to get maximum deflections. This ensured an increase in thermal gradient, maintaining a thermophoretic force that could drive particles in the desired pathway within the lattice. The thermophoretic coefficients were adjusted for different particle sizes in correspondence with experimental observations.

## Online content

Any methods, additional references, Nature Portfolio reporting summaries, source data, extended data, supplementary information, acknowledgements, peer review information; details of author contributions and competing interests; and statements of data and code availability are available at 10.1038/s41566-025-01731-z.

## Supplementary information

Supplementary Information Supplementary Figs. 1–10 and Discussion.

Supplementary Video 1 Engineered gap in the thermal barrier leads to particle crossing: when a gap in the thermal profile is created (Supplementary Fig. 3b), particles can cross the barrier, visible as particle defocusing. Video size: 100 × 100 μm.

Supplementary Video 2 Moving particles avoiding an optofluidic barrier: the presence of a horizontal optofluidic barrier (Fig. 1j) in the centre forces sedimenting particles (*d*_p_ = 5 μm) to move around it. Video size: 100 × 100 μm.

Supplementary Video 3 A tilted optofluidic barrier enables particle steering: for a –45°-tilted barrier (Fig. 2a), particles (*d*_p_ = 5 μm) are being steered towards the right. Video size: 100 × 100 μm.

Supplementary Video 4 A splitter barrier design divides the particle flow into two: for two tilted barriers connected at the top (Fig. 2b), particles (*d*_p_ = 5 μm) are being steered left and right, effectively splitting the flux of particles equally into two. Video size: 100 × 100 μm.

Supplementary Video 5 A merger barrier design focuses particle flow in the centre: for two barriers tilted towards the bottom with a 20-μm gap (Fig. 2c), particles (*d*_p_ = 5 μm) are being concentrated in the centre. Video size: 100 × 100 μm.

Supplementary Video 6 A U-shaped barrier allows particle trapping: side-by-side video of two U-shaped barriers of two laser powers, leading to the stable trapping of a single particle at different equilibrium positions (Fig. 2d). Total input laser power, *P*: 95 mW (left) and 135 mW (right). Video size: 40 × 40 μm.

Supplementary Video 7 Periodic switching for a collective of particles: the orientation of an optofluidic barrier of the steerer type is periodically switched each 30 s (Fig. 3a). Video size: 100 × 100 μm.

Supplementary Video 8 Feedback-induced switching for a single particle: the orientation of an optofluidic barrier of the steerer type is switched based on the particle’s position to follow a targeted path (Fig. 3b). Video size: 20 × 50 μm.

Supplementary Video 9 Shifting the trapping position through the reconfiguration of a U-shaped barrier: side-by-side view of two methods of shifting the equilibrium position of a single particle in a U-shaped barrier over time. Left: by switching the input laser power (*P* = 95 mW and *P* = 135 mW; Supplementary Video 6), the position is shifted vertically (Fig. 3d,e). Right: by laterally moving the U-shaped barrier, the particle position follows horizontally (Fig. 3c). Video size: 40 × 50 μm.

## Source data

Source Data Fig. 1 Statistical source data.

Source Data Fig. 2 Statistical source data.

Source Data Fig. 3 Statistical source data.

Source Data Fig. 4 Statistical source data.

## Data Availability

Data for Figs. 1–4 are available via Zenodo at 10.5281/zenodo.13990372 (refs. ^39–41^). Source data are provided with this paper.
