# Supplementary material for: Three-dimensional optofluidic control using reconfigurable thermal barriers
Source: Nat Photonics. 2025 Aug 8;19(12):1385–91. doi: 10.1038/s41566-025-01731-z (PMC12672375; doi:10.1038/s41566-025-01731-z)
Supplement: Supplementary file 1 — Supplementary Figs. 1–10 and Discussion. [file 41566_2025_1731_MOESM1_ESM.pdf]

nature photonics

Supplementary information https://doi.org/10.1038/s41566-025-01731-z

Three-dimensional optofluidic control using reconfigurable thermal barriers

In the format provided by the authors and unedited

Supplementary Information

Heat-induced phenomena in liquids

Local light absorption creates strong temperature gradients (∇T) near the surface, which alter fluid properties such as density, permittivity, and entropy. This drives thermoosmotic and convective fluid flows, as well as the thermophoretic movement of particles. Below, we briefly outline the origin of these flows, their specific roles and compare the results from experiments and simulations (Fig. S1).

Thermoosmosis occurs when temperature gradients perturb interfacial interactions (e.g., van der Waals forces and the electric double layer) at the liquid-solid boundary [18]. Strong interfacial flows emerge in the presence of steep temperature gradients, dragging particles along and enabling precise manipulation. Their strength depends on the involved materials and the strength of ∇T. For the latter, in typical microfluidic settings, the in-plane gradients are determined by the extent and strength of the heat sources (Fig. 1c), while out-of-plane gradients also depend on the heat flux along z given its much smaller dimension (here $h = 20\,\mu$m). Therefore, the thermal conductivity κ of the boundaries matters, and can vary strongly between materials ($\kappa_{\text{glass}} = 1.2\,\text{WK}^{-1}\text{m}^{-1}$, $\kappa_{\text{PDMS}} = 0.15\,\text{WK}^{-1}\text{m}^{-1}$) influencing the thermal gradient as previously reported [27]. In our experiments we reach temperature gradients up to $2.6\,\text{K}\mu\text{m}^{-1}$ resulting into flow speeds of $v_{\text{TO}}|_{r=2\,\mu\text{m}} = 22\,\mu\text{ms}^{-1}$, which confirms previous observations [18]. Thermoosmosis can also be modifying the ion concentration in solution does altering the van der Waals interaction [18], rendering it a highly tunable flow field.

Natural convection results from local changes in fluid density under ∇T. In our system, thermoosmotic flows trigger the formation of upward-directed convective patterns, driven by the temperature gradient. These patterns, similar in shape to thermoosmotic flows, form closed loops, although they extend beyond our field of view. As shown before, they mostly depend on the thickness of the microfluidic chamber (Fig. S2) and assist in steering particles away from the barrier when those are being injected from the top either by sedimentation (Figs. 1-4) or by pressure-driven flows (Fig. S5).

Thermophoresis refers to the movement of particles along a temperature gradient, influenced by changes in interfacial interactions between the particle and fluid, which depends on the surface charge of the particle and any ion concentration in solution affecting the interaction strength. The direction of motion can vary, but in most synthetic systems, including ours, colloids exhibit thermophobic behaviour, moving away from hot regions [34]. From our decomposition of thermal effects observed in the flow profiles around a single heat source (Fig. S1) we can determine the thermophoretic mobility D_T by using $v_{\text{TP}} = -D_T \frac{dT}{dr}$, with v_{TP} the thermophoretic velocity, and $\frac{dT}{dr}$ the temperature gradients (Fig. 1b,c). We find $D_T = 8.7\,\mu\text{ms}^{-1}\text{K}^{-1}$, which agrees with previous experimental observations for silica particles [23, 40]. In contrast, some biological organisms, such as cells, display thermophilic behaviour in strong temperature gradients, which has been used to trap them in focused laser beams. This behaviour may arise from changes in water permittivity and the electric double layer near the surface of the charged cell [19].

Thermoviscous Flows While in principle also changes in the liquid viscosity under ∇T could play a role, the induced heat sources remain mostly stationary in our experiments, such that thermoviscous flows can be neglected [20].
The interplay of these thermal effects varies depending on the substrate, particle type, and chamber thickness, allowing for fine-tuning of each parameter.
All thermal effects play crucial roles in forming optofluidic barriers, as their overall outward-directed flows ($z = 5-15\,\mu$m for $h = 20\,\mu$m) push particles away from the barrier, preventing passage. Simulations further reveal that the thermophobic behaviour of the particles enhances this effect, as they consistently move away from hot regions, reinforcing the barrier's interaction with them.

Non-thermal contributions

Among the non-thermal contribution, only sedimentation due to density mismatches of particles and medium and optical forces could contribute. While we have investigated the latter in detail in a previous study [28], in particular scattering and gradient forces, here, we ensured that the focus of the laser beam was situated slightly outside the microfluidic chamber reducing optical forces to a minimum.
However, the sedimentation of our silica particles plays a significant role in our system given its vertical orientation. The sedimentation velocity can be determined with the Stokes law [30] using

$$v_g = \frac{d_\mathrm{p}^2(\rho_\mathrm{p} - \rho_\mathrm{m})g}{18\eta}, \tag{S1}$$

with particle density $\rho_\mathrm{p} = 1850\,\mathrm{kgm}^{-3}$, water density $\rho_\mathrm{m} = 980\,\mathrm{kgm}^{-3}$ and viscosity $\eta = 0.93\,\mathrm{mPs}$ at $T = 23°\mathrm{C}$, and g the gravitational constant. While $v_g < 1.5\,\mu\mathrm{ms}^{-1}$ is negligible for particles with $d_\mathrm{p} < 1.5\,\mu$m, for particles used in Figs. 2-4 with $d_\mathrm{p} > 4\,\mu$m it becomes considerable with $v_g > 8\,\mu\mathrm{ms}^{-1}$.

DLD

Additional simulations were conducted to gain a deeper understanding of the behaviour exhibited by the DLD assay. To that end, we run simulations with a modified heat delivery scheme, wherein selective increases in heat were applied to specific pillars (see Fig. S8). As mentioned in the main text, we observed that if we deliver more heat in certain pillars in order to increase the thermal gradient, which provides a greater thermophoretic force, we could achieve better deflection efficiencies.
Further, we conducted simulations to explore the range from $dx = 21\ \mu$m to $dx = 34\ \mu$m, and with a distinct dy value ($dy = 25\ \mu$m) (see Fig. S9). Firstly, it was determined that the value of dx can be adjusted to filter a specific particle size. In our case, $dx \in [21\ \mu\mathrm{m}, 22\ \mu\mathrm{m}]$ is adequate to deflect 4 μm particles, while $dx \in [29\ \mu\mathrm{m}, 34\ \mu\mathrm{m}]$ is adequate to preferentially deflect 6 μm particles. Both particle sizes are deflected when $dx \in [22\ \mu\mathrm{m}, 29\ \mu\mathrm{m}]$. Notably, as we increase dx, the deflection efficiency of 4 μm particles decreases. Also, it was discovered that if dy is smaller than a certain threshold, the thermal gradient is insufficient to develop the thermophoretic force necessary to deflect the particles.

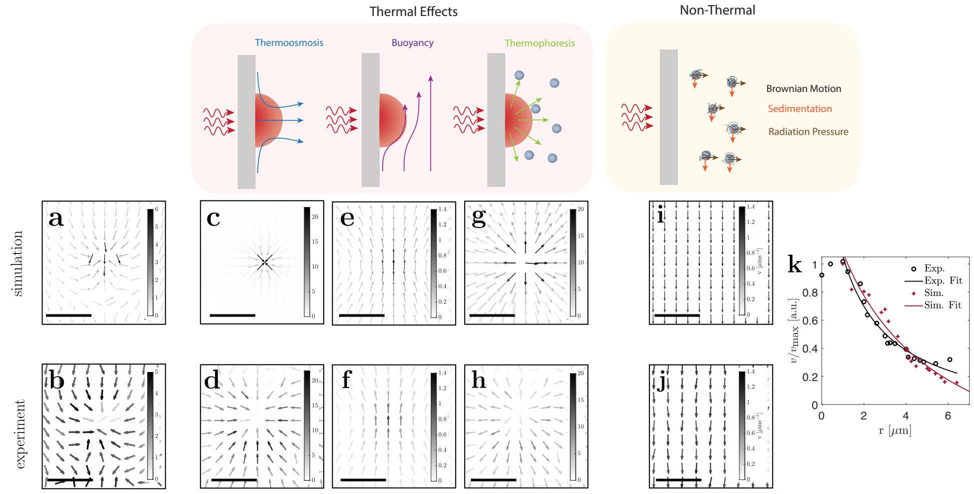

Fig. S1 Contribution of thermal and non-thermal effects on the observed flow fields around a single heat source with $\Delta T_{\max} = 20\,\mathrm{K}$. **a,b** Total observed flow fields near the surface at $z = 2\,\mu$m in simulations and experiments, respectively. **c,e,g,i** Simulated flow fields for individual contributions, and **d,f,h,j** their respective decomposition in experiments. In simulations, the thermal effects **c** thermoosmosis, **e** natural convection, and **g** thermophoresis are modeled separately. Experimentally, the decomposition is performed by first considering **b** the total flow velocity map and subtracting contributions from **f** natural convection, as determined from simulations, and **i,j** sedimentation, as given by Eqn. S1. The relative contributions of simulated **c** thermoosmotic and **g** thermophoretic effects to the total simulated flow field are then used to estimate the experimental **d** thermoosmotic and **h** thermophoretic contributions. This estimation uses the known characteristic flow directions for our system, where thermoosmotic flow is radially inward and thermophoretic flow radially outward. **k** Comparison of normalized velocity $v/v_{\max}$ taken from **a,b** as a function of radial distance r. The fitted curves follow $\sim r^{-1}$ dependence, demonstrating good agreement between experimental and simulated results. Scale bars 25 μm.

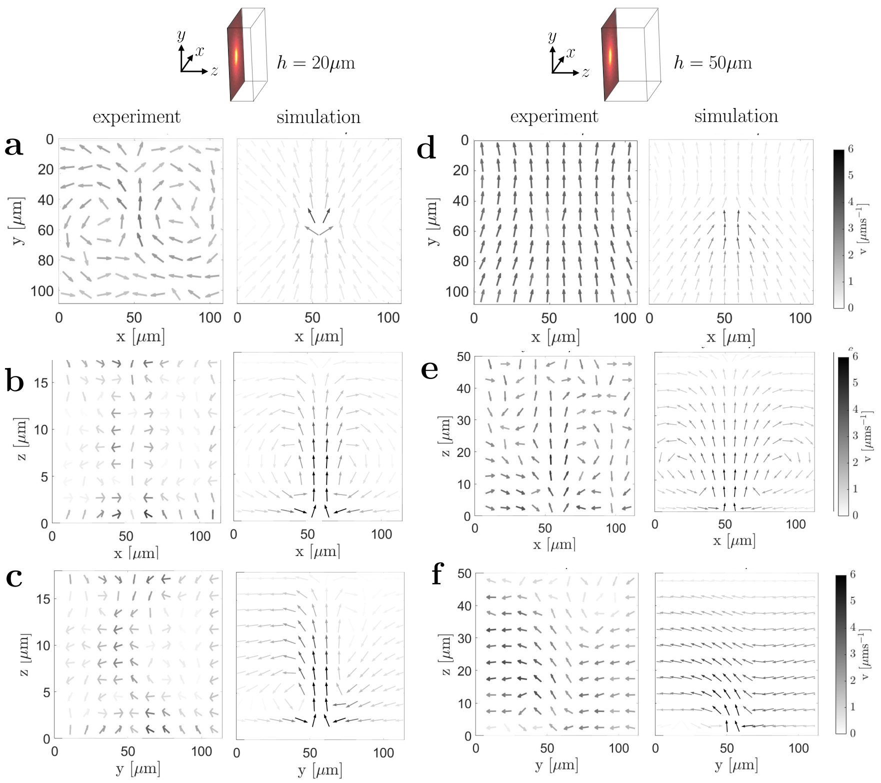

Fig. S2 Effect of chamber thickness h on convective flows measured experimentally (left) and in simulations (right) in the middle of the chamber $z = h/2$ for single heat source with $\Delta T_{\max} = 25\,$K. **a-c** For chambers with $h = 20\ \mu$m, **a** natural convection becomes only apparent for $z > 10\,\mu$m with the typical closed-loop appearance, **b** while thermo-osmotic flows are still the dominant driver of fluid flows orthogonally, **c** and parallel to the axis of gravity y. **d-f** As the thickness increases to $h = 50\ \mu$m, **d** natural convection is pronounced, visible by the increasing upwards bias. **e** While the thermoosmotic flows remain constant, **f** natural convection is overtaking the system's flow dynamics.

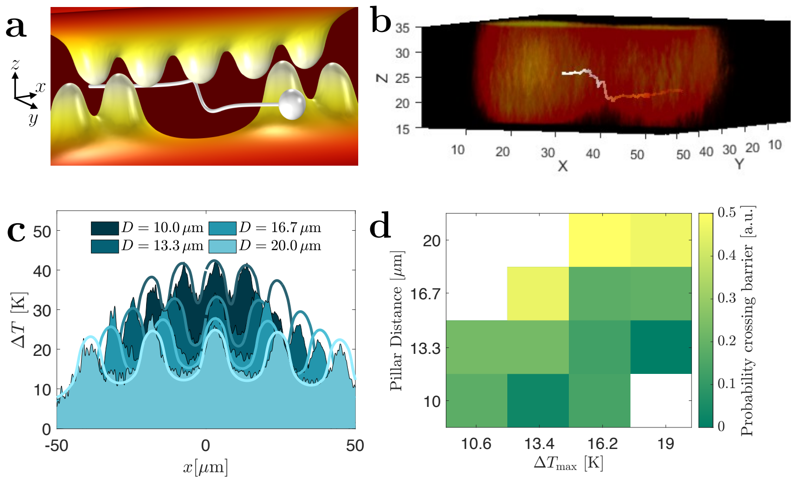

Fig. S3 Performance of optofluidic barrier. a,b A gap is created by omitting two pillars on one side, allowing sedimenting particles ($d_\mathrm{p} = 5\,\mu$m) to pass through the barrier by changing their z position, **a** in simulations and **b** in experiments. This passage is observed in video S1 as a defocusing of the particle near the barrier, indicating its movement through the gap. **c-d** By changing the thermal landscape, the barrier's performance is modified. **c** The spatial cross-section of the temperature profile shows increasing gap sizes between pillars as the separation distance increases, with experimental data (filled plot) and simulations (line) in agreement. In experiments, particles exhibit similar behaviour, shifting their z-position and passing through the bottom gap. **d** Quantitative analysis of the particle crossing probability as a function of maximum temperature increase ΔT_max and pillar separation distance. The barrier performs optimally at higher ΔT_max and smaller pillar distance, where particle passage is most effectively blocked.

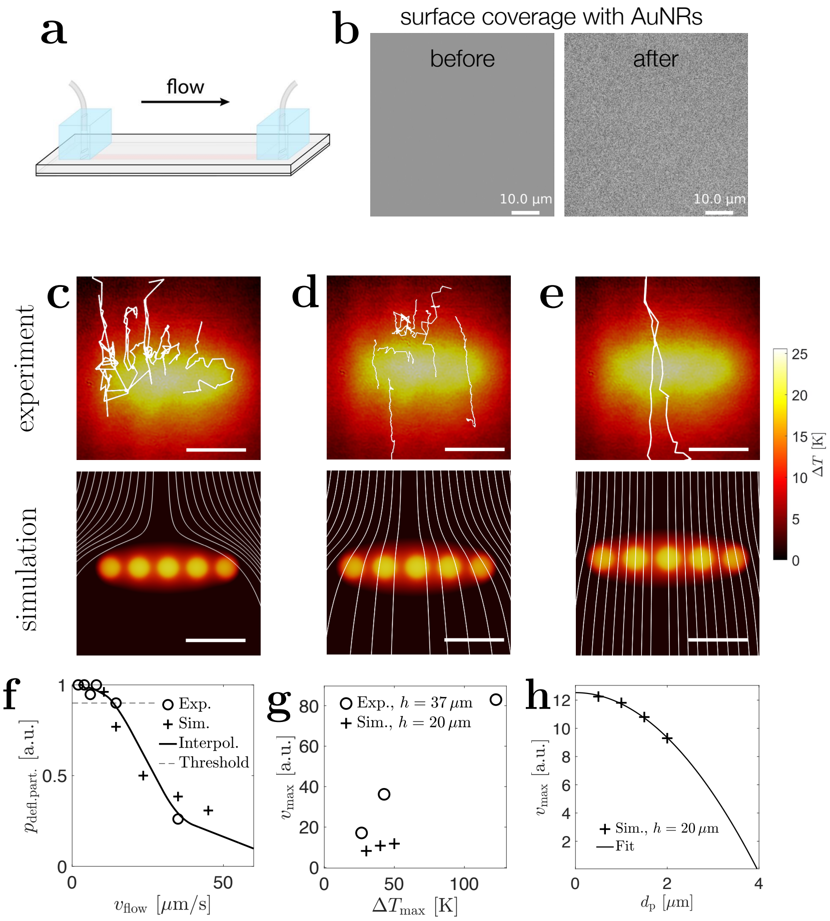

Fig. S4 Performance of optofluidic barriers in pressure-driven microfluidics. a Sketch of a single flow channel embedded in a glass chip. In- and outlets are connected to PDMS blocks through which the tubing to a pressure pump could be attached. **b** Before and after deposition of AuNRs onto the glass surfaces measured with a label-free interferometric setup exhibiting strong scattering from the deposited particles [41]. **c-e** Trajectories (white) of silica particles $d_p = 1.5\,\mu$m in the presence of a barrier within a channel of $h = 6\,\mu$m; with flow speeds **c** $v_{\text{flow}} = 10\,\mu\text{ms}^{-1}$, **d** $v_{\text{flow}} = 24\,\mu\text{ms}^{-1}$, and **e** $v_{\text{flow}} = 45\,\mu\text{ms}^{-1}$. **f** Barrier performance as ratio of deflected particles $p_{\text{defl.part.}}$ under varying pressure-driven flows v_{flow} for experiments (circles) and simulations (crosses), where we define the barrier functional until a maximum flow speed v_{max} for deflection efficiencies $\geq$ 90% (dashed line) through interpolation (continuous line). **g** Maximum applicable flow speed v_{max} depending on maximum temperature increase ΔT_{max} for experiments with a chamber height of $h = 37\,\mu$m, and simulations $h = 20\,\mu$m. **h** v_{max} is reduced by the sedimentation velocity v_g (Eqn. S1) with increasing d_{p} at fixed $\Delta T_{\text{max}} = 25\,$K. Scale bars $10\,\mu$m.

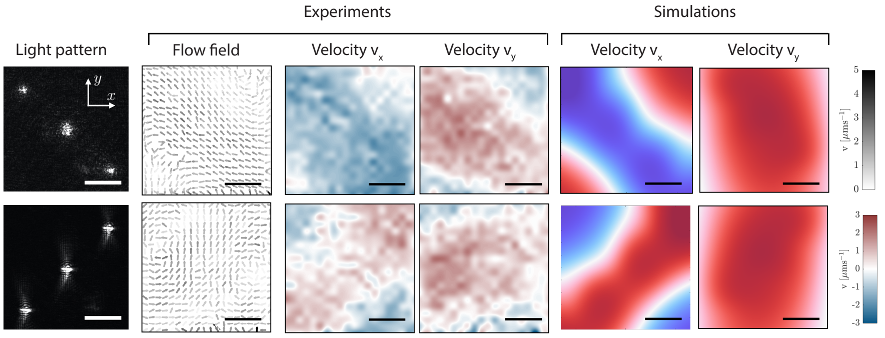

Fig. S5 Optofluidic actuators driven by convective patterns. In a $h = 50\ \mu$m thick chamber, natural convection is predominantly generated using single light spots as localized heat sources (white laser beam reflections from the glass slide), guiding particles in the direction of the heat patterns. Shown here are two different tilt orientations (top: towards up left, bottom: towards up right). The flow fields were measured by tracing silica particles ($d_\mathrm{p} = 1.5\,\mu$m) indicating the direction of overall flows. The velocity component v_x illustrates change in flow direction for both tilts (negative velocities in blue show left movement, while positive velocities in red show right movement). Given the larger h, natural convection flows become predominant as seen for velocity v_y. Experiments and simulations show a good agreement of flow directionality depending on induced light patterns. Scale bars 15 μm.

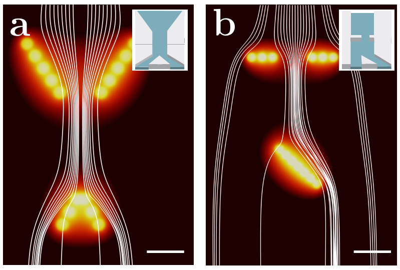

Fig. S6 Combining multiple types of barriers into a single workflow. Using the basic optofluidic barriers from Figs. 1-2, we demonstrate the creation of more complex flow environments to replicate common microfluidic tasks. **a** A design with a narrowing channel and two outlets, where streams are first focused and then split. **b** A configuration that first selects particles using a slit, then separates them from the main channel. Scale bars 20 μm.

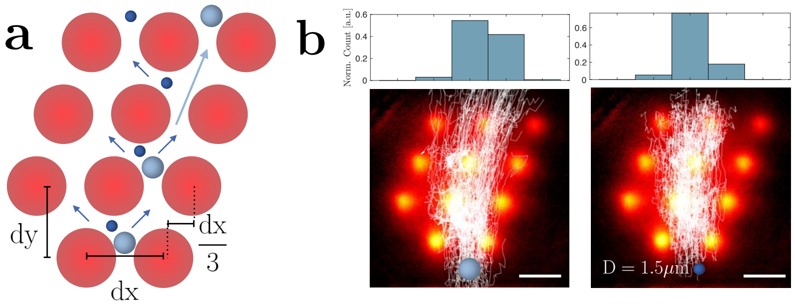

Fig. S7 Additional DLD experiments. **a** For small particles ($d_\mathrm{p} < 3\ \mu$m), buoyancy dominates over sedimentation, driving the particle upwards and thus requiring the DLD pattern to be reversed for separation. **b** Effective separation occurs in a mixture of $d_\mathrm{p} = 3\ \mu$m and $d_\mathrm{p} = 1.5\ \mu$m particles: larger particles are displaced to the right (40%), while smaller ones mostly travel straight through with minimal displacement. Scale bars 10 μm.

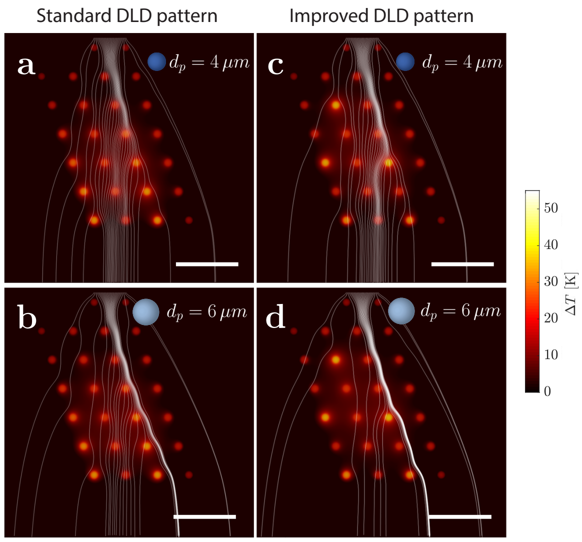

Fig. S8 Enhanced separation efficiency in DLD. Compared to the standard DLD patterns shown in Fig. 5 (left), improvements were made in simulations by selectively heating individual pillars. This strategic modification enhances the separation of $d_{\mathrm{p}} = 6$ μm from $d_{\mathrm{p}} = 4$ μm, since the former group is deflected more effectively. Scale bar 25 μm.

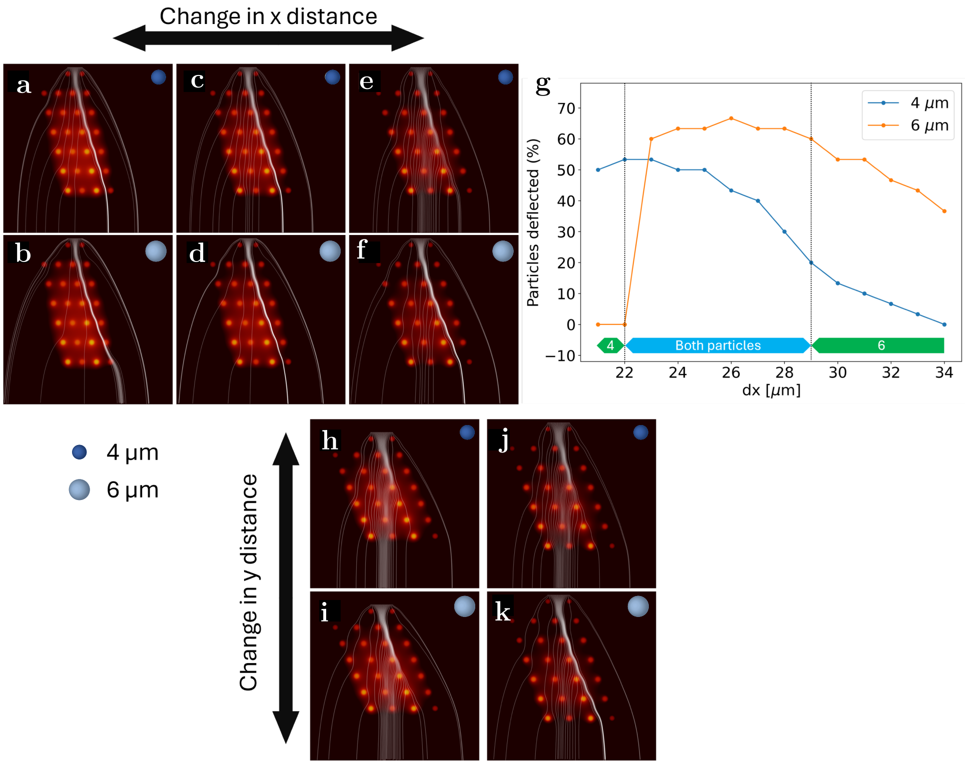

Fig. S9 DLD separation efficiency dependence on pillar spacing. Simulated temperature profiles of the DLD assay for different dx distances, **a,b** $dx = 22\ \mu$m, **c,d** $dx = 25\ \mu$m and (**e,f**) $dx = 30\ \mu$m, and particles trajectories for $d_\mathrm{p} = 4\ \mu m$ (**a,c,e**) and $D = 6\ \mu m$ **b,d,f**, respectively. (**g**) Percentage of deflected particles as a function of dx. For $dx \in [21\ \mu\text{m}, 22\ \mu\text{m}]$, only $d_\mathrm{p} = 4\ \mu$m particles are deflected **a,b**, while $d_\mathrm{p} = 6\ \mu$m particles are preferentially deflected for $dx \in [29\ \mu\text{m}, 34\ \mu m]$ **e,f**. Both particle sizes are deflected when $dx \in [23\ \mu\text{m}, 29\ \mu m]$ **c,d**. In this range, as dx increases, the deflection efficiency is reduced for $d_\mathrm{p} = 4\ \mu$m particles. We also analyze the change in distance along y, $dy = 25\ \mu$m **h,i**, and **j,k** for $dy = 30\ \mu$m. Scale bar 25 μm.

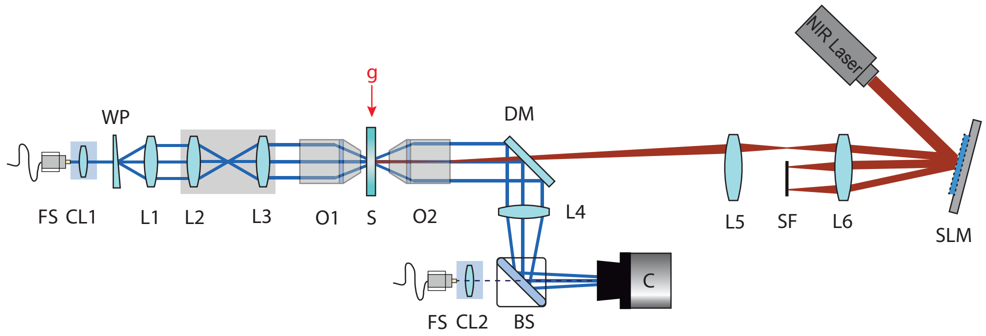

Fig. S10 Experimental Setup. For the probe beam, a high-power fiber laser for illumination ($\lambda = 465\,\text{nm}$, LaserTack) is coupled into the setup through two fiber splitters (FS). After collimation (CL1), the illumination beam is passed through a wedge-prism with 2° deviation (WP, Thorlabs PS810) mounted on a rotation stage (Thorlabs PRM1). After passing through a beam expander (L1/L2) the beam is relayed to a long working distance objective (O1, Olympus LMPLFLN 50x/0.50 NA) illuminating a vertically mounted sample (S) with indicated direction of gravity g. The forward scattered light is collected by an objective (O2, Olympus UPLANFL 40x/0.75NA), reflected by a dichroic mirror (DM), and focused by a lens (L4) onto the CMOS camera (C, Basler acA1920-155um). The second illumination path is collected by (CL2), passing through a 90:10 R:T beam splitter (BS, Thorlabs BSX16) and projected onto the camera. For the pump beam, a NIR laser ($\lambda = 808\,\text{nm}$, Roithner Laser Technik RLTMDL-808) is expanded and projected onto a spatial light modulator (SLM, Hamamatsu SLM LCOS-SLM IR -02). In a 4f-configuration (L5/L6) 0-th order is spatially filtered out (SF) before being projected through a DM and onto the sample via O2.
